# Carvedilol Exerts Cardioprotective Effects Against Doxorubicin Toxicity via Autophagy Modulation and Energetics Restoration

**DOI:** 10.3390/ph19060845

**Published:** 2026-05-28

**Authors:** Asma Boukhalfa, Pei-Tsz Shin, Dawn M. Meola, Ada Yu, Amene Majidipur, Annie Showers, Dylan A. Valencia, Emmanuella F. Akomeah-Sirleaf, Jenica N. Upshaw, Cheryl A. London, Iris Z. Jaffe, David E. Sosnovik, Lakshmi Pulakat, Vicky K. Yang, Howard H. Chen

**Affiliations:** 1 Molecular Cardiology Research Institute, Tufts Medical Center, Boston, MA 02111, USA; 2 Cummings School of Veterinary Medicine, Tufts University, North Grafton, MA 01536, USA; 3 School of Medicine, Tufts University, Boston, MA 02111, USA; 4 Division of Cardiology, Tufts Medical Center, Boston, MA 02111, USA; 5 Cardiovascular Research Center, Massachusetts General Hospital, Harvard Medical School, Charlestown, MA 02129, USA

**Keywords:** cardio-oncology, chemotherapy-induced cardiotoxicity, cardioprotective agents, molecular imaging, autophagy, biomarkers

## Abstract

**Background/Objectives**: Carvedilol is an adrenergic blocker FDA-approved to improve outcomes in heart failure with reduced ejection fraction. Clinical trials examining whether carvedilol may be cardioprotective in the setting of cancer therapy-induced heart failure have generated mixed results that may depend on the cancer regimen, tumor, or comorbidities. **Methods**: To investigate the therapeutic potential of carvedilol to mitigate doxorubicin cardiotoxicity in cardiomyocytes, myocardial tissue, and in vivo, independent of confounding factors in clinical studies, we utilized disease-free cardiac slices and cardiomyocytes from mice, dogs, and human in vitro, and in wildtype mice injected with doxorubicin in vivo. We further evaluated the impact of carvedilol in dogs with cancer receiving doxorubicin. **Results**: In primary canine and murine cardiac slices, carvedilol treatment restored autophagy and prevented apoptosis from doxorubicin. Carvedilol restored mitochondrial energetics in human, canine, and murine models. In wildtype mice challenged with doxorubicin, carvedilol prevented declines in cardiac function and alterations in cardiac structure. In pet dogs with cancer and undergoing doxorubicin treatment, carvedilol was beneficial in preserving cardiac function and structure. **Conclusions**: Carvedilol activates cardioprotective autophagy, arrests doxorubicin-induced cell death, and improves energetics and cardiac structure and function across species.

## 1. Introduction

Cardiovascular disease is the second leading cause of mortality among cancer survivors, and in many patients with cancer, the risk of cardiovascular death is higher than the risk of death from cancer [1]. A growing number of cancer treatments, including chemotherapeutics and targeted treatments, have been associated with cardiomyopathy and heart failure. Anthracyclines, including doxorubicin, are topoisomerase II and mTOR inhibitors that remain as the first line therapy to treat many solid and hematologic malignancies, despite their well-established risk for cardiotoxicity. Even when managed carefully, there is no safe dosage of doxorubicin known to be completely risk-free for heart failure during or after cancer treatment.

Currently the only FDA-approved drug designated to prevent anthracycline-induced cardiotoxicity, dexrazoxane, is not routinely prescribed in clinical settings due to the pleiotropic effects of the drug and concerns about cancer efficacy and potential toxicity [2]. There is thus an urgent and unmet clinical need to develop new interventions that have clear molecular mechanisms to exert cardioprotective effects amenable for translation. Carvedilol is an FDA-approved adrenergic blocker widely used to treat heart disease. Clinical trials examining the efficacy of carvedilol to treat doxorubicin-induced cardiotoxicity have generated conflicting results [3,4,5,6], suggesting that further preclinical and translational research is warranted.

Autophagy is an evolutionarily conserved process that is central to maintaining cellular homeostasis by eliminating potentially harmful stressors from the cell [7,8]. In cardiomyocytes, autophagy removes damaged mitochondria, a highly proapoptotic stimulus, from the cells and enhances cardiomyocyte survival after stress [9,10]. Cardiomyocyte autophagy can be dysregulated by chemotherapy, and in that setting, autophagy restoration is cardioprotective [11,12,13,14]. Thus, we tested the hypothesis that carvedilol activates cardiomyocyte autophagy to exert cardioprotective effects during doxorubicin exposure. Using a combination of ex vivo myocardial slice cultures, in vitro cardiomyocytes, an in vivo mouse model, and canines with cancer, we explored the pathophysiological impact of carvedilol on autophagy, cell fate, and cardiac function in the setting of doxorubicin-induced cardiotoxicity.

## 2. Results

### 2.1. Cardiac Protective Autophagy Restoration in Canine and Murine Cardiac Slices

**Figure 1:**
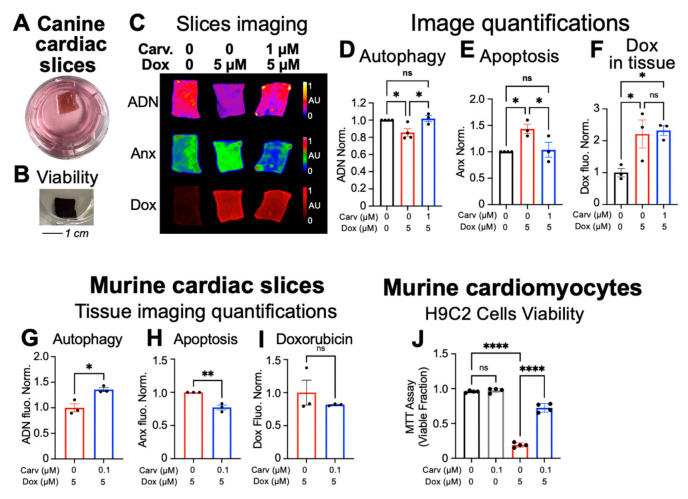
Carvedilol pre-treatment restored doxorubicin-induced autophagy impairment and reduced apoptosis in canine and murine hearts. (**A**) Image of a canine cardiac slice cultured in transwell. (**B**) Viability of cardiac slice confirmed by MTT staining. (**C**) Fluorescence reflectance imaging of autophagy with autophagy detecting nanoparticle (ADN) [12], apoptosis with fluorescent Annexin V (Anx), and doxorubicin (Dox) uptake and retention in live canine cardiac slices, and the quantifications (**D**–**F**). (**G**–**I**) Quantification of ADN (autophagy), fluorescent Annexin V (apoptosis), and doxorubicin uptake and retention in live mouse cardiac slices challenged with 5 μM Dox for 24 h with or without pre-treatment with carvedilol for 4 h. (**J**) Quantification of MTT assay of cell viability in rat H9C2 cardiomyocytes that are either untreated, incubated with 5 μM Dox alone, or 0.1 μM of carvedilol alone, or pre-treated with carvedilol before Dox. Multiple comparisons comparing the mean of each column with the mean of every other column were conducted. n = 3 biological replicates for canine and murine slices (**C**–**I**); n = 4 biological replicates for H9C2 cells (**J**). Relevant comparisons but not all significant changes are shown. ns = not significant, * *p* < 0.05, ** *p* < 0.01, **** *p* < 0.0001, ANOVA with TUKEY post-test.

An example of a canine cardiac slice is shown in Figure 1A. Once tissue viability was confirmed by MTT (Figure 1B), slices were challenged with 5 μM doxorubicin with or without 1 μM carvedilol pretreatment. Direct live tissue imaging demonstrated that doxorubicin (Dox) significantly reduced autophagy levels compared to untreated control (Figure 1C,D). This Dox-induced impairment of cardiac autophagy was restored to basal levels by pretreatment of 1 μM of carvedilol (Carv). The autophagy restoration by carvedilol correlated with a significant decrease in apoptosis, as quantified by Annexin V staining (Figure 1C,E). Imaging of inherent doxorubicin fluorescence suggested that carvedilol pretreatment did not significantly alter doxorubicin uptake or retention in the myocardium (Figure 1F). In mouse cardiac slices exposed to doxorubicin with or without carvedilol pretreatment, carvedilol significantly increased ADN signal and decreased Annexin V fluorescence (Figure 1G,H), consistent with the autophagy stimulation and apoptosis reduction seen in the canine cardiac slices. Carvedilol did not alter doxorubicin uptake and retention compared to doxorubicin-only controls (Figure 1I). In H9C2 rat cardiomyocytes, carvedilol significantly improved cell survival in the presence of doxorubicin (Figure 1J).

### 2.2. Carvedilol Improves Energetics Across Species

**Figure 2:**
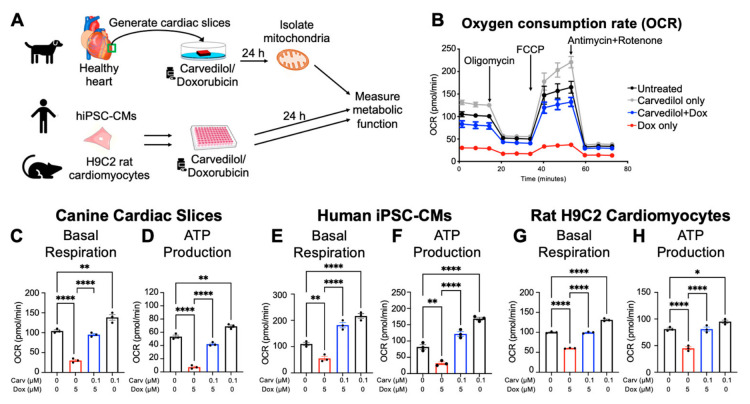
Carvedilol improved mitochondrial energetics in canine hearts and in human and murine cardiomyocytes. (**A**) Experimental outline. (**B**) Representative mitochondrial respiration graphs. (**C**,**D**) Quantification of basal respiration and ATP production by Seahorse assay of isolated mitochondria from canine cardiac slices that are either untreated, incubated with 5 μM of doxorubicin (Dox) alone, 0.1 μM of carvedilol (Carv) alone, or pre-treated with 0.1 μM of carvedilol before Dox. (**E**,**F**) Quantification of Seahorse assay of basal respiration and ATP production in human iPSC-CMs that are either untreated, incubated with 5 μM Dox alone, or 0.1 μM of carvedilol alone, or pre-treated with carvedilol before Dox. (**G**,**H**) Quantification of Seahorse assay of basal respiration and ATP production in rat H9C2 cardiomyocytes that are either untreated, incubated with 5 μM Dox alone, or 0.1 μM of carvedilol alone, or pre-treated with carvedilol before Dox. n = 3 biological replicates for canine cardiac slices (**C**,**D**), for human IPSC-CMs (**E**,**F**), and for H9C2 cells (**G**,**H**). Multiple comparisons comparing the mean of each column with the mean of every other column were conducted. Relevant comparisons but not all significant changes are shown. ns = not significant, * *p* < 0.05, ** *p* < 0.01, **** *p* < 0.0001, ANOVA with TUKEY post-test.

To investigate the functional impact of carvedilol-mediated autophagy restoration, we assessed mitochondrial energetics (Figure 2A,B). In mitochondria isolated from the canine cardiac slices, doxorubicin alone significantly reduced basal respiration and ATP production compared to untreated controls (Figure 2C,D). Carvedilol treatment significantly improved doxorubicin-impaired basal respiration and ATP production to a level comparable to the untreated controls. In both human iPSC-derived cardiomyocytes (iPSC-CMs) and H9C2 rat cardiomyocytes, doxorubicin similarly impaired basal respiration and ATP production compared to untreated controls (Figure 2E–H), both of which were significantly rescued by carvedilol. The cross-species rescue of basal respiration and ATP production by carvedilol confirms the direct protective effect of carvedilol on at-risk cardiomyocytes during doxorubicin stress.

### 2.3. Carvedilol Exerts Cardiac Protective Effects in Mice In Vivo

**Figure 3:**
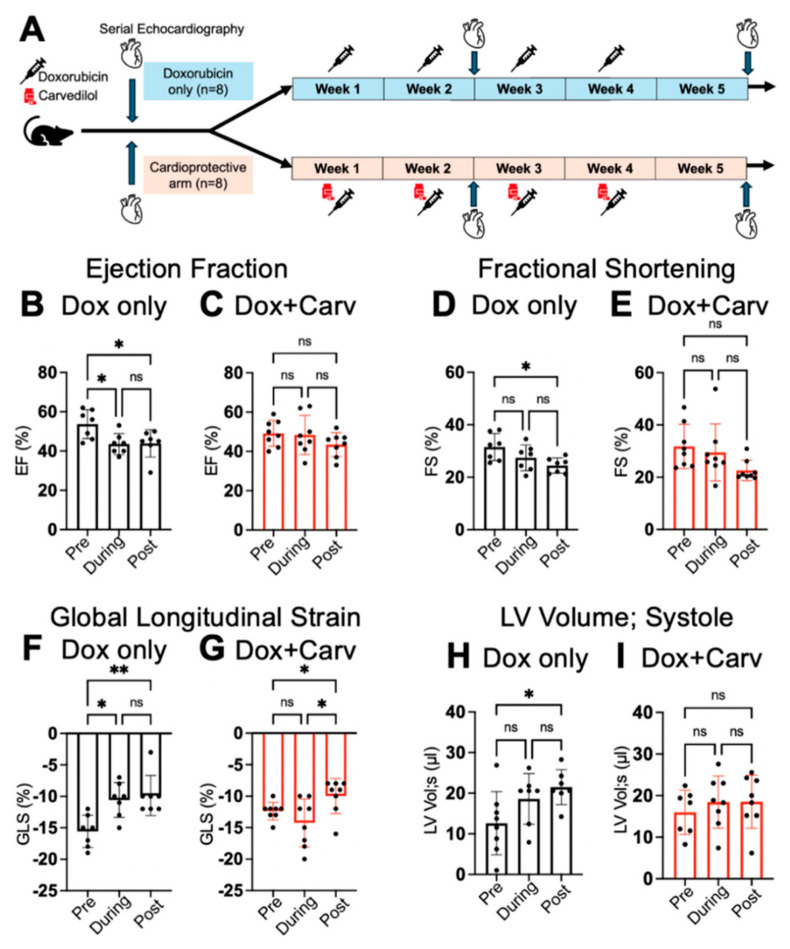
Cardioprotective effects of carvedilol in naive mice challenged with doxorubicin. (**A**) Schematic of naive mice challenged with doxorubicin (Dox) with or without carvedilol pretreatment (Carv). (**B**,**C**) Serial echocardiograph assessment of cardiac function by ejection fraction (EF) before given doxorubicin with or without carvedilol pretreatment (Pre), immediately before the third dose of carvedilol by oral gavage and one day before doxorubicin injection (During), or one week after the final and fourth cycle of doxorubicin (Post). (**D**,**E**) Echocardiograph measures of fractional shortening (FS). (**F**,**G**) Echocardiograph measures of Global Longitudinal Strain (GLS). (**H**,**I**) Echocardiograph measures of left ventricular volume in systole (LV Vol;s). n = 8 mice/biological replicates for DOX only, and n = 8 mice/biological replicates for Dox+Carv. ns = not significant, * *p* < 0.05, ** *p* < 0.01, ANOVA with TUKEY post-test.

To investigate the effect of carvedilol pretreatment in vivo, C57Bl/6J wildtype mice (n = 16) were either given doxorubicin alone (weekly intravenous injection for four cycles) or received carvedilol the day prior to each doxorubicin injection (Figure 3A). Cardiac function was examined by echocardiography during treatment, the day before the third doxorubicin dose, and post treatment at one week after the fourth doxorubicin dose (Figure 3A and Supplementary Table S1). Ejection fraction (EF, Figure 3B,C) and fractional shortening (FS, Figure 3D,E) were significantly reduced by doxorubicin during and post treatment, which were rescued in mice pretreated with carvedilol. Global longitudinal strain (GLS) was significantly affected in mice during and post doxorubicin administration, and the decrease in strain defect was rescued by carvedilol during treatment (Figure 3F,G). Left ventricular (LV) volume significantly increased post doxorubicin treatment, which was not seen with carvedilol pretreatment (Figure 3H,I).

### 2.4. Carvedilol with Lisinopril Exerts Cardiac Protective Effects in Canines with Cancer

**Figure 4:**
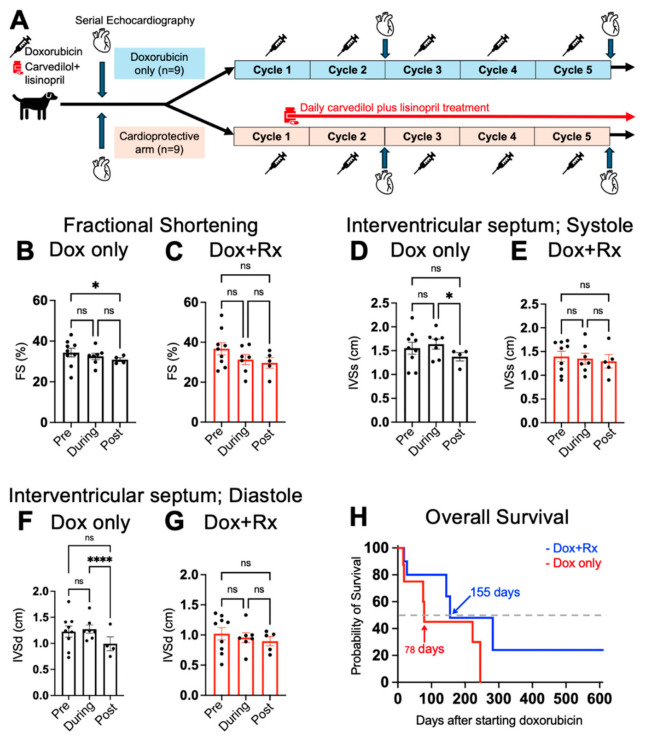
Cardioprotective effects of carvedilol plus lisinopril in canine patients treated with doxorubicin. (**A**) Schematic of canines with cancer treated with doxorubicin (Dox) with or without cardioprotective carvedilol plus lisinopril intervention (Rx). (**B**,**C**) Serial echocardiograph assessment of cardiac function by fractional shortening (FS) before starting doxorubicin/carvedilol+lisinopril regimen (Pre), immediately before the third cycle of doxorubicin (During), or one month after finishing the five cycles of doxorubicin (Post). (**D**,**E**) Echocardiograph measures of interventricular septum thickness in systole (IVSs). (**F**,**G**) Echocardiograph measures of interventricular septum thickness in diastole (IVSd). (**H**) Kaplan–Meier survival curve annotated with the 50% survival (gray dashed line) and median survival (arrows). n = 9 dogs/biological replicates for doxorubicin only, and n = 9 dogs/biological replicates for the doxorubicin/carvedilol+lisinopril cardioprotective arm. ns = not significant, * *p* < 0.05, **** *p* < 0.0001, ANOVA with TUKEY post-test.

We next investigated the therapeutic potential of carvedilol pretreatment in dogs with spontaneous hemangiosarcoma, a cancer similar to angiosarcoma in humans [15]. Eighteen dogs undergoing cancer treatment were enrolled in a prospective randomized clinical trial to test the cardioprotective effects of carvedilol with lisinopril, the standard of care in the veterinary clinic, during and after cardiotoxic doxorubicin treatment (Figure 4A and Supplementary Table S2). Canines with hemangiosarcoma and receiving doxorubicin treatment, intravenously every three weeks for a total of five doses, were randomized into cardioprotective (receiving oral carvedilol and lisinopril daily, n = 9) or control (placebo, n = 9) groups. Fractional shortening was assessed during treatment, right before the third doxorubicin dose, and after treatment with cycle five, and was significantly reduced after doxorubicin treatment (Figure 4B). This decline in fractional shortening was not statistically significant in the cardioprotective cohort (Figure 4C). Interventricular septum thickness during systole (Figure 4D,E) and diastole (Figure 4F,G) was significantly reduced by doxorubicin. These myocardial structural alterations were not observed in canine patients in the cardioprotective arm. While the overall survival was not significantly different between the two groups (n = 9 in each cohort), median survival was 155 days after starting doxorubicin for the cardioprotective group compared to 78 days without carvedilol (Figure 4H).

## 3. Discussion

We have shown that carvedilol restores autophagy and exerts anti-apoptotic effects (Figure 1) and restores mitochondrial respiration (Figure 2) in mouse and dog cardiac tissues ex vivo and in rat and human cardiomyocytes in vitro. Carvedilol given to wildtype mice prior to doxorubicin administration boosted autophagy and preserved cardiac function and structure (Figure 3). Moreover, carvedilol prevented the decline in cardiac function observed during and post doxorubicin treatment (Figure 4B,C). It is intriguing that a single dose of carvedilol given the day before doxorubicin administration in mice was sufficient to prevent cardiac dysfunction during and after repeated doxorubicin injections. This supports the notion that an acute boost of autophagy can allow the rapid degradation and recycling of mitochondria during subsequent stress conditions or disease states.

The pleiotropic effect of carvedilol is often ascribed to its beta-blocking, vasodilatory, and antioxidant properties, all of which may exert protective effects on the heart. For example, beta-receptor blockade may lead to a decrease in myocardial oxygen consumption, especially important during periods of stress and disease [16]. As such, carvedilol is part of the treatment regimen for heart failure with reduced ejection fraction, and clinical trials of carvedilol in this population have demonstrated improved survival, reduced hospitalization, and improved LVEF [17]. However, the effect of decreased myocardial oxygen consumption is likely less pronounced in a cultured non-beating cardiac slice. Carvedilol has also been reported to exert antioxidant and cardioprotective effects in human failing hearts [18]. At the molecular level, carvedilol has been known to stimulate the beta-arrestin-mediated EGFR transactivation pathway to confer these protective effects [19,20]. In cardiomyocytes, carvedilol can also stimulate AMP-activated protein kinase (AMPK) and inhibit the mammalian target or rapamycin (mTOR) pathway or phosphorylate Unc-51 like autophagy activating kinases (ULK) 1 and 2 and Beclin 1, upregulating autophagy [21,22,23,24]. Doxorubicin on the other hand inhibits AMPK phosphorylation and downstream autophagy activation [25], and is further shown to interfere with autophagosome fusion with lysosomes, reducing autophagic flux [13,26]. Reactivating autophagy in turn has been shown to protect cardiomyocytes and the heart from doxorubicin [12,13,27]. This is consistent with our findings that in both murine and canine cardiac slices, carvedilol restores beneficial autophagy, detectable by the autophagy-detecting nanoparticle, in the presence of doxorubicin without affecting doxorubicin fluorescence in myocardial tissue (Figure 1).

Our results demonstrated that mitochondrial production of ATP can be restored with carvedilol, and this effect is consistent across species (Figure 2), suggesting an evolutionarily conserved cardiomyocyte survival mechanism in the presence of doxorubicin. In addition to targeting topoisomerase II, doxorubicin can damage mitochondrial membrane lipids, resulting in reduction in ATP production, mitochondrial stress, and the generation of reactive oxygen species (ROS). ROS can further impair mitochondrial function reducing ATP production, and contribute to the accumulation of detrimental mitochondrial mutation [28,29,30]. Carvedilol can scavenge ROS and reduce oxidative stress [31,32,33,34], and activate AMPK, a key mediator of cellular responses to energetic stress and mitochondrial insults. This raises intriguing possibilities that mitophagy, an autophagic process that removes damage mitochondrial, may play a role in restoring cardiomyocyte energetics to exert cardioprotective effects, and thus warrants deeper mechanistic investigations [35,36]. The multi-faceted cardioprotective effects seen with carvedilol can be further investigated with additional beta- and alpha-blockers to determine any class or compound specific effects, and further in combination with additional cardioprotective agents to maximize the beneficial effects on the heart that will be amendable for translation.

Development of a cardioprotective regimen during chemotherapy is of great importance as risk of developing cardiovascular side effects is high. However, the incorporation of carvedilol as part of the cardioprotective protocols in the cardio-oncology clinic has yet to be determined. Numerous clinical trials in human patients have sought to determine the effects of carvedilol on echocardiographic changes and clinical outcome after anthracycline therapy. The results are however mixed. A meta-analysis of six trials showed that, compared to placebo, carvedilol did not prevent the decrease in LVEF, but at the same time, fewer clinically overt cardiotoxicity events were recorded. These studies were short, following the patients only up to 6 months while demonstrating great heterogeneity between trials [37,38]. Separately, the OVERCOME trial showed preservation of left ventricular ejection fraction with the combination of carvedilol and enalapril after 6 months of follow-up [39], but the CECCY trial failed to show maintenance of the cardiac systolic function with carvedilol alone, but rather a rescue of the cardiac diastolic function [4,40]. Because of these conflicting results, ongoing trials with longer follow-up periods are being conducted; administering carvedilol at different dosages in adults and in pediatric patients, in addition to trials of other classes of drugs (e.g., statins, ACE inhibitors, SGLT2 inhibitors) is much needed to provide viable clinical guidelines to prevent or treat cardiovascular dysfunction in cancer patients [38].

Pet dogs that spontaneously develop cancer and undergo cancer treatment represent a uniquely suitable clinical model to investigate cancer treatment-induced cardiotoxicity and cardioprotective interventions. Trials using pet dogs that have shorter life spans compared to humans allow for shorter clinical studies and relatively lower cost compared to human clinical trials. Our pilot canine trial demonstrated the potential beneficial effects of carvedilol in preserving cardiac structure and function during and post doxorubicin regimen without adversely affecting median survival time (Figure 4). This pilot study has a small sample size, and outcome may be confounded by different stages of cancer and concurrent administration of other medications such as lisinopril. Nevertheless, cardiac function maintenance in the patients given carvedilol and lisinopril is consistent with the autophagy restoration and anti-apoptotic effects specifically attributed to carvedilol and seen in cancer-free canine cardiac slices challenged with doxorubicin (Figure 1). However, we cannot rule out the possibility that carvedilol can slow heart rate and reduce cardiac workload, which may contribute to the cardioprotective effects we see in vivo. Furthermore, whether carvedilol is given after the first dose of doxorubicin as in the caner dogs, or given 24 h before each doxorubicin instillation as in our mouse study, carvedilol similarly exerted beneficial effects on cardiac function (Figure 3 and Figure 4). These promising results suggest that a larger prospective study in animals or humans is warranted to optimize the treatment protocol that would maximize the cardioprotective potential of carvedilol in cardio-oncology.

There is currently a lack of suitable early biomarkers for chemotherapy-induced cardiotoxicity to monitor the response to cardioprotective intervention [41,42,43,44]. We demonstrate here the ability to directly quantify autophagy levels in live tissue in real time (Figure 1). In both canine and murine cardiac slices, autophagy dysregulation due to doxorubicin was restored by carvedilol. Autophagy imaging may represent a novel biomarker to monitor cardiotoxicity when autophagy is dysregulated and when autophagy is normalized with successful implementation of cardioprotection to guide therapy [12]. We provide here a platform for future mechanistic studies of autophagy signaling needed to optimize the treatment of cancer drugs associated cardiotoxicity.

Limitations of Research: All dogs used for the cardiac slices are males due to the availability of tissue. We were thus unable to delineate any sex differences in those experiments. In the in vivo mouse study, we included four females and four males in each of the doxorubicin-only or the cardioprotective arms. For the in vivo cancer dog study, while the cardioprotective arm includes five females and four males, the placebo cohort consists of one female and eight males due to the randomization protocol of the study. We found no apparent sex difference, presumably due to sample size, and thus reported pooled data of both sexes. Furthermore, the mix of dog breeds pooled for the study all contributes to the interindividual variabilities seen even at baseline. A larger clinical trial will be needed to elucidate any underlying sex and age differences in the response to carvedilol. Secondly, the use of carvedilol in combination with lisinopril, an ACE (Angiotensin-Converting Enzyme) inhibitor, which is the standard of care in the veterinary clinic, confounds the effects of carvedilol alone. Further detailed mechanistic studies are thus needed to decipher the isolated as well as the combination effects of carvedilol and lisinopril on cardiomyocytes and the myocardium. Thirdly, subjects in the canine clinical study received additional treatments, including pain and sedation mediations, GI protectant and steroids, as deemed necessary by the attending oncologists. As the disease progresses, dog owners can eventually elect euthanasia if there is a perceived decrease in quality of life, affecting the survival time reported in our study.

## 4. Materials and Methods

Sex as a biological variable: Our study examined both male and female animals, and combined findings from both sexes are reported.

Canine cardiac slice culture: Adult canine hearts were collected from heart disease and cancer free dogs whose owners elected euthanasia at the Tufts University Foster Hospital for Small Animals and consented through the tissue donation program at Tufts University Cummings School of Veterinary Medicine. The tissue donation program has received Tufts Institutional Animal Care and Use Committee (IACUC) exemption and does not require IACUC review and approval. The demographic information of these dogs is shown in Table 1. Humane euthanasia was performed by the attending veterinarian according to standard protocols with the administration of commercially available Euthasol consisting of pentobarbital sodium and phenytoin sodium at 1 mL per 4.5 kg body weight. To ensure cardiomyocytes viability and metabolic function, each heart was harvested within 10 min of euthanasia and kept in cold (4 °C) sterile cardioplegic solution (5.5 mM glucose, 0.5 mM MgSO_4_, 24 mM KCl, 15 mM NaHCO_3_, 109 mM NaCl, 0.9 mM NaH_2_PO_4_, 1.8 mM CaCl_2_, pH 7.4) for 24 h. Transmural sections of the LV free wall measuring 1 cm × 1 cm were excised. These tissue blocks were then sectioned using a vibrating microtome (Campden Model 5100 mz, Campden Instruments, Leicestershire, UK) in cold oxygenated slicing solution (30 mM 2,3-butanedione monoxime, 1 mM glucose, 10 mM HEPES, 6 mM KCl, 140 mM NaCl, 1 mM MgCl_2_, 1.8 mM CaCl_2_, pH 7.4) as previously described. A total of 13 resulting slices of 350 μm thickness were washed, placed on a 0.3 μm pore size transwell surface in 6-well culture plates, and cultured in media (M199 with 0.1% BSA, 1× ITS, 10 mM 2,3-butanedione monoxime, 1× chemically defined lipid, 100 U/mL penicillin/streptomycin, 1× HEPES) at the air–media interface at 37 °C with 5% CO_2_.

Mouse cardiac slice culture. A total of 5 (2 female and 3 male) C57BL/6J mice (Jackson Laboratory, Bar Harbor, ME, USA, stock# 000664) at 20 weeks of age were combined for the study since no gender-specific effects were apparent. Mice were euthanized using CO_2_, and each heart was harvested immediately after euthanasia and kept in cold (4 °C) sterile slicing solution for immediate slicing using a similar procedure to the one described above with the following differences: short-axis slices of 300 μm thickness were generated and cultured in Claycomb media (Sigma-Aldrich, St. Louis, MO, USA) with 100 μM norepinephrine, 10% fetal bovine serum, and 4 mM L-glutamine at the air–media interface on 0.3 μm pore-size transwell at 37 °C with 2% CO_2_.

Both canine and mouse cardiac slice viability was confirmed by 3-(4,5-dimethylthiazol-2-yl)-2,5-diphenyltetrazolium bromide (MTT) staining as previously described [11]. The viability test was repeated daily on untreated cardiac slices to ensure maintenance of viability in culture. To minimize any between-group variations and increase the robustness of our findings, slices were then randomized [45] to either left untreated as control, treated with 0.1 μM or 1 μM of carvedilol (Tokyo Chemical Industry, Tokyo, Japan, C226) alone for 24 h, or pretreated for 4 h before a 24 h exposure to 5 μM of doxorubicin (Cayman Chemical, Ann Arbor, MI, USA, 15007). The carvedilol dosing was chosen based on the reported carvedilol peak plasma concentration of 0.48 μM in humans [46], modeling the effect seen in the myocardium when administered clinically. The 5 μM doxorubicin dosing was chosen to induce cardiomyocyte apoptosis in vitro [12].

Imaging of cardiac slices. At 4 h before the treatment endpoint, Autophagy Detecting Nanoparticle (ADN) [12], a fluorescently-activatable nanoprobe that tracks autophagosome fusion and degradation in autophagolysosomes, was added at 10 μg Fe/mL. Fluorescent Annexin V (PerkinElmer, Waltham, MI, USA) to stain for apoptosis was added 1 h before imaging. The slices were then washed with 1× cold PBS, and fluorescence reflectance imaging of ADN (675 nm excitation/720 nm emission), Annexin (745 nm excitation/800 nm emission) and doxorubicin (535 nm excitation/600 nm emission) was performed on IVIS spectrum (Perkin Elmer). Average fluorescence intensity was performed across the entire surface area for each cardiac slice. Fluorescence intensity was then normalized to the untreated slices that were included in each imaging session as control. All image quantifications were performed using ImageJ (Version 1.53n) (NIH).

Cell culture. H9C2 rat cardiomyoblasts (ATCC, CRL-1446) were cultured in Dulbecco’s Modified Eagle’s Medium supplemented with 10% fetal bovine serum. iCell human cardiomyocytes differentiated from human induced pluripotent cells (iPSCs) (Fujifilm, Cellular Dynamics, Madison, WI, USA) were thawed and handled according to manufacturer’s instructions. For the Seahorse assay, either H9C2 cells or iCells were counted and plated on 96-well microplates at 20,000 cells/well. Cells were then allowed to settle and cultured in a 37 °C incubator with 5% CO_2_. After culturing (24 h for H9C2 cells, 7 days for iCells), cells were either left untreated, or treated with 0.1 μM of carvedilol for 24 h with or without 5 μM of doxorubicin. Doxorubicin was added to culture for 30 min and subsequently replaced with fresh complete media to mimic the clinical scenario of rapid doxorubicin washout from circulation. MTT staining to assess H9C2 cardiomyocytes viability was performed as previously described [12].

Oxygen consumption rate (OCR). Cardiac slices in buffer (10 mM HEPES, 220 mM Mannitol, 70 mM Sucrose, 1 mM EGTA, 0.5 mM phenylmethylsulfonyl fluoride, pH 7.4) were minced, homogenized, and centrifuged (700 g, 10 min, 4 °C) to collect the supernatant. After centrifugation (10,000 g, 10 min, 4 °C), the mitochondria pellet was washed once and resuspended in buffer (70 mM sucrose, 220 mM mannitol, 10 mM KH_2_PO_4_, 5 mM MgCl_2_, 1 mM EGTA, 2 mM HEPES, pH 7.4) for protein quantification. Mitostress assay with 600 ng of mitochondria or 20,000 cells was performed according to the manufacturer’s instructions. The basal oxygen consumption was measured in the presence of Complex I substrate (5.5 mM Malate, 5.5 mM Pyruvate and 2.2 mM ADP) followed by sequential addition of the mitochondrial assay solution (control), or 25 µL of 45 µmol/L Oligomycin, 10 µmol/L FCCP, 55 µmol/L Antimycin and 55 µmol/L Rotenone. ATP production rate was derived by isolating the ATP-linked component of the OCR traces, added to the glycolytic ATP production calculated from the proton efflux. Both OCR and ATP production were quantified and normalized to mitochondrial protein content (for cardiac slices) or cell number (for iCells and H9c2 cardiomyocytes) per the manufacturers’ instructions on the Seahorse XF96 analyzer (Agilent, Santa Clara, CA, USA).

Mouse study in vivo. C57BL/6J mice were purchased from the Jackson Laboratory (stock#000664). A total of 16 (n = 8 each of female and male) mice between 8 and 10 weeks of age were randomized to either receive doxorubicin only (doxorubicin 5 mg/kg, by intravenous injection, weekly for 4 consecutive weeks, n = 8) or receive carvedilol (5 mg/kg, by oral gavage, n = 8) 24 h prior to doxorubicin injections. All mice were maintained on 12 h alternating light and dark cycles with free access to water and food (Teklad 2916 irradiated diet, Envigo, Indianapolis, IN, USA). To assess cardiac structure and function changes, transthoracic echocardiographic imaging was performed with a 13.0 MHz linear probe (Vevo 2100, VisualSonics, Toronto, ON, Canada) and analyzed by 2 experienced echocardiographers blinded to the experimental design, as previously described [12].

Canine cancer patient study. The dogs (Table 2) were required to be at least 1 year of age and have undergone splenectomy and histological confirmation of the diagnosis of splenic hemangiosarcoma. Exclusion criteria included evidence of significant abnormalities in the baseline complete blood count, biochemistry panel or urinalysis, or pre-existing cardiac disease. The doxorubicin dose was determined by the attending oncologist, at 30 mg/m^2^ intravenously for dogs >10 kg, or 1 mg/kg intravenously for dogs ≤10 kg, within the accepted published dose range for each animal. The dogs were randomized 1:1 to either the control arm (no additional cardiac medications given) or the cardioprotective arm, receiving 0.5 mg/kg carvedilol and 0.5 mg/kg lisinopril, both orally, twice a day.

Echocardiogram was performed at baseline (prior to doxorubicin treatment initiation), prior to the third doxorubicin dose, and one month after completing doxorubicin treatment (total of five doses), and every three months thereafter until the animals reached the endpoint of the study. Study endpoints included recurrence of neoplasia or clinical signs associated with hemangiosarcoma, requiring cardiac medication for arrhythmia control, or death.

Echocardiographic images (GE Vivid E95/E9) were obtained by a board-certified veterinary cardiologist. Echocardiographic exams included standard 2-dimensional and M-mode images, and LVEF by Simpson’s method of discs. All echocardiographic measurements were performed by the same observer. M-mode measurements were normalized to patient body weight using previously published formulas [47].

Statistics. Statistical analysis of the data and the generation of graphs were performed using Prism 10 (GraphPad). All data were tested for normality using the D’Agostino–Pearson omnibus normality test, the Shapiro–Wilk test and the Kolmogorov–Smirnov test. No outlier detection was performed due to the relatively small sample size of our studies. Instead, manual inspection of our dataset was performed to identify any obvious experimental or data entry error. For all tissue imaging, protein blot, and Seahorse assay data, comparison between two groups was performed with a two-tailed unpaired *t*-test if normal, and a Mann–Whitney test if non-Gaussian. Analysis of variance (ANOVA) was used for comparisons of three or more groups, followed by Tukey post-test. All echocardiography data, which was acquired serially, was analyzed using One-Way ANOVA for repeated measures data with a mixed-effects model that uses the maximum likelihood method with Geisser–Greenhouse correction. Multiple comparisons comparing the mean of each column with the mean of every other column were corrected with Tukey’s multiple comparisons test, with individual variances computed for each comparison. Kaplan–Meier survival curve was generated and analyzed in Prism. The data in the figures are graphed using dot plots, with the average and standard error bars indicated. For all assays, experiments were performed in at least three biologically independent samples. The differences between groups were considered statistically significant at *p* < 0.05.

## Figures and Tables

**Table 1:** Demographic information of the dogs euthanized for cardiac slices.

Breed	Age (Years)	Sex	Weight (kg)
Mix breed	13	Male castrated	28.0
Jack Russell	13	Male castrated	6.3
Mix breed	3	Male castrated	25.3
Dachshund	3	Male castrated	10.0
Shepherd Cross	7	Male castrated	20.0
Pit Bull	3	Male intact	35.0

**Table 2:** Demographic information of the hemangiosarcoma canine patients undergoing doxorubicin treatment and were enrolled in the carvedilol cardioprotection canine trial.

Breed	Age (Years)	Sex	Weight (kg)	Treatment Arm
Mixed breed	11	Male castrated	21.0	Placebo
Mixed breed	9	Male castrated	41.7	Cardioprotective
Chesapeake Bay Retriever	9	Male castrated	38.0	Placebo
Mixed breed	10	Male castrated	13.5	Cardioprotective
Border Terrier	10	Female spayed	8.9	Cardioprotective
Labrador Retriever	11	Male castrated	33.0	Placebo
Mixed breed	9	Female spayed	23.2	Cardioprotective
Mixed breed	11	Male castrated	28.5	Placebo
Gordon Setter	11	Female spayed	28.5	Cardioprotective
German Shorthair Pointer	8	Male castrated	27.0	Placebo
German Shepherd	9	Male castrated	45.7	Placebo
Bichon Frise	9	Male castrated	10.3	Placebo
Shih Tzu	11	Male castrated	10.1	Cardioprotective
Mixed Breed	7	Male castrated	35.0	Cardioprotective
Mixed breed	10	Female intact	26.4	Placebo
Mixed breed	11	Female spayed	21.4	Cardioprotective
German Shepherd	11	Male castrated	40.0	Placebo
Mixed breed	11	Female spayed	13.7	Cardioprotective

## Data Availability

The datasets generated and analyzed during the current study are available from the corresponding authors upon reasonable request.

## Supplementary Materials

The following supporting information can be downloaded at: https://www.mdpi.com/article/10.3390/ph19060845/s1, Table S1: Echocardiographic data for mice in vivo study; Table S2: Echocardiographic data for canine cancer patients.

## Abbreviations

The following abbreviations are used in this manuscript:

FDA	Food and Drug Administration
mTOR	Mammalian Target of Rapamycin
MTT	3-(4,5-dimethylthiazol-2-yl)-2,5-diphenyltetrazolium bromide
Dox	Doxorubicin
Carv	Carvedilol
ADN	Autophagy Detecting Nanoparticle
ATP	Adenosine Triphosphate
iPSC-CMs	Human iPSC-derived cardiomyocytes
EF	Ejection Fraction
FS	Fractional Shortening
GLS	Global Longitudinal Strain
LV	Left Ventricular
EGFR	Epidermal Growth Factor Receptor
AMPK	AMP-Activated Protein Kinase
IACUC	Institutional Animal Care and Use Committee
OCR	Oxygen Consumption Rate
ANOVA	Analysis of Variance
LV Vol;s	Left Ventricular Volume in Systole
IVSs	Interventricular Septum Thickness in Systole
IVSd	Interventricular Septum Thickness in Diastole
