# Supplementary material for: Carvedilol Exerts Cardioprotective Effects Against Doxorubicin Toxicity via Autophagy Modulation and Energetics Restoration
Source: Pharmaceuticals (Basel). 2026 May 28;19(6):845. doi: 10.3390/ph19060845 (PMC13304947; doi:10.3390/ph19060845)
Supplement: Supplementary file 1 [file pharmaceuticals-19-00845-s001.zip › pharmaceuticals-4299372-supplementary.pdf]

Supplementary Table S1. Echocardiographic data for mice *in vivo* study

	Doxorubicin Only		Carvedilol + Doxorubicin	
	**Mean**	**STD**	**Mean**	**STD**
**EF (%) pre**	53.71	7.319	49.25	6.563
**EF (%) during**	43.57	5.318	48.38	9.956
**EF (%) post**	43.86	6.986	43.50	6.071
**FS (%) pre**	31.50	5.160	31.79	8.439
**FS (%) during**	27.38	4.913	29.46	10.880
**FS (%) post**	24.45	2.900	22.60	3.902
**GLS (%) pre**	-15.57	2.573	-12.38	1.408
**GLS (%) during**	-10.57	2.760	-14.25	3.808
**GLS (%) post**	-9.86	3.185	-10.00	2.777
**LV volume in systole (µl) pre**	12.59	7.782	15.99	5.316
**LV volume in systole (µl) during**	18.58	6.219	18.46	6.298
**LV volume in systole (µl) post**	21.47	4.299	18.54	6.377

Supplementary Table S2. Echocardiographic data for canine cancer patients

	Doxorubicin Only		Carvedilol/Lisinopril + Doxorubicin	
	**Mean**	**STD**	**Mean**	**STD**
**FS (%) pre (Cycle 1)**	34.35	6.418	36.73	9.107
**FS (%) during (Cycle 3)**	32.46	4.129	31.33	6.614
**FS (%) post (1 month post)**	30.87	1.894	29.67	5.968
**IVSs (cm) pre (Cycle 1)**	1.552	0.3772	1.396	0.3312
**IVSs (cm) during (Cycle 3)**	1.636	0.2761	1.350	0.3060
**IVSs (cm) post (1 month post)**	1.373	0.1773	1.294	0.3198
**IVSd (cm) pre (Cycle 1)**	1.227	0.3236	1.020	0.3097
**IVSd (cm) during (Cycle 3)**	1.273	0.2101	0.957	0.2138
**IVSd (cm) post (1 month post)**	0.993	0.2656	0.898	0.1720
